# Optimal ranking and directional signature classification using the integral strategy of multi-objective optimization-based association rule mining of multi-omics data

**DOI:** 10.3389/fbinf.2023.1182176

**Published:** 2023-07-27

**Authors:** Saurav Mallik, Soumita Seth, Amalendu Si, Tapas Bhadra, Zhongming Zhao

**Affiliations:** ^1^ Environmental Health, Harvard T. H. Chan School of Public Health, Boston, MA, United States; ^2^ Center for Precision Health, School of Biomedical Informatics, The University of Texas Health Science Center at Houston, Houston, TX, United States; ^3^ Department of Computer Science and Engineering, Brainware University, Kolkata, India; ^4^ Department of Computer Science and Engineering, Aliah University, Kolkata, India; ^5^ School of Information Technology, Maulana Abul Kalam Azad University of Technology, Haringhata, India; ^6^ Human Genetics Center, School of Public Health, The University of Texas Health Science Center at Houston, Houston, TX, United States

**Keywords:** multi-objective optimization, distance-based dynamic support threshold, multi-objective optimized association rule mining, multi-omics sarcoma data, empirical Bayes test

## Abstract

**Introduction:** Association rule mining (ARM) is a powerful tool for exploring the informative relationships among multiple items (genes) in any dataset. The main problem of ARM is that it generates many rules containing different rule-informative values, which becomes a challenge for the user to choose the effective rules. In addition, few works have been performed on the integration of multiple biological datasets and variable cutoff values in ARM.

**Methods:** To solve all these problems, in this article, we developed a novel framework *MOOVARM* (multi-objective optimized variable cutoff-based association rule mining) for multi-omics profiles.

**Results:** In this regard, we identified the positive ideal solution (*PIS*), which maximized the profit and minimized the loss, and negative ideal solution (*NIS*), which minimized the profit and maximized the loss for all gene sets (item sets), belonging to each extracted rule. Thereafter, we computed the distance (*d* +) from PIS and distance (*d* −) from NIS for each gene set or product. These two distances played an important role in determining the optimized associations among various pairs of genes in the multi-omics dataset. We then globally estimated the relative closeness to *PIS* for ranking the gene sets. When the relative closeness score of the rule is greater than or equal to the pre-defined threshold value, the rule can be considered a final resultant rule. Moreover, *MOOVARM* evaluated the relative score of the rule based on the status of all genes instead of individual genes.

**Conclusions:** *MOOVARM* produced the final rank of the extracted (multi-objective optimized) rules of correlated genes which had better disease classification than the state-of-the-art algorithms on gene signature identification.

## 1 Introduction

A microarray (Bandyopadhyay et al., 2014; Bandyopadhyay and Mallik, 2016) has been widely used to measure a large number of genes for determining differences between two groups (e.g., cases *versus* control samples), including gene expression profile, methylation, and genotype-based association studies. Methylation of cytosine (Navarro et al., 2012) changes the structure of *DNA* by introducing a methyl group (−*CH*3) at the carbon5 position of cytosine without altering the underlying *DNA* sequences. Methylation changes the gene expression, which pathologically leads to cancer. In general, methylation decreases the gene expression level. At present, the association rule mining (*ARM*) method plays a vital role in generating the significant relationships between two genes (items) in the research field of bioinformatics and biomedical sciences (Mallik, 2013). The rule representing the format is as follows: {*G*1+, *G*2−, *G*4+ ⇒ *G*3−, *G*5+ }, where *G*1, *G*2, and *G*4 are the cause variables (antecedent) and *G*3 and *G*5 are effective variables (consequent). Of note, here, “+” symbolizes upregulation and “−” denotes downregulation. The aforementioned rule states that when the genes *G*1 is upregulated, *G*2 is downregulated, and *G*4 is upregulated concurrently, it is likely that *G*3 will be downregulated and *G*5 will be upregulated simultaneously. The support of a rule {*A* ⇒ *B*} (where *A* and *B* are items) is defined as the fraction of the number of transactions that contains *A* and *B* to the total number of transactions in the database, whereas the confidence of the rule is defined as the ratio of the support of the whole gene set (i.e., *A* and *B*) to the support of the antecedent/left-hand side (i.e., *A*). If the support of the gene set is higher than the user-defined minimum support, then the gene set is called frequent. A useful and fundamental association rule mining method, Apriori, was introduced by Agrawal et al. (1993) for identifying the association among the genes in the gene expression data or other similar kinds of data. Apriori and Eclat are the two benchmark algorithms used for mining the frequent item sets. The Apriori algorithm is introduced by Agrawal et al. (1993), while Eclat was developed by Zaki (2000) (Alves et al., 2010). The basic steps of the Apriori algorithm are as follows: i) obtaining the support (frequency) value for each individual item (feature), ii) filtering out non-frequent items by using a user-defined support threshold, iii) selecting frequent k-item sets (*k* = 1,2,×), iv) then converting all frequent item sets into association rules, and v) finally, estimating two more rule interestingness measures, *viz.*, confidence and lift. However, the Eclat algorithm (Zaki, 2000) is somewhat different from Apriori (Agrawal et al., 1993). Apriori is basically a join-based algorithm, while Eclat is a tree-based algorithm. In other words, Apriori follows a breadth-first search (horizontal search), while Eclat follows a depth-first search (vertical search). Eclat is faster than Apriori. The Eclat algorithm requires only the support metric. Both the algorithms use static support and static confidence thresholds.

This basic *ARM* method has been updated and modified depending on the problem types to overcome various limitations by the researchers, such as Han et al. (2004), Creighton and Hanash (2003), Georgii et al. (2005), McIntosh and Chawla (2007), and Martinez et al. (2008). Those updated techniques help us manage the critical problems which arise in our daily life like medical diagnosis, marketing, and traveling. The genes of the gene sets have different types of priority. However, the basic rule mining algorithms treat all genes of the gene sets as belonging to the same class equivalence (quality). To overcome this challenge, the following researchers introduced weighted *ARM* methods for the classification of genes: Ramkumar (1998), Cai (1998), Wang (2000), Tao (2003), Yun and Leggett (2005), Tseng (2010), and Mallik et al. (2015). The weighted *ARM* methods were further modified and considered multiple weighted factors for solving transaction data-related problems (Liu et al., 1999; Su et al., 2008; Liu et al., 2011). Some clustering- and biclustering-based techniques were invented for studying gene expression data by Cheng and Church (2000), Pei (2003), Jiang et al. (2004), Madeira and Oliveira (2004), Thalamuthu et al. (2006), and Prelic et al. (2006). StatBicRM (Maulik et al., 2015), another classification analysis, was also developed for this reason, in which Bhasin and Raghava (2004), Paziewska et al. (2014), Martella (2009), Liu and Xu (2009), and Georgii et al. (2005) used a half-space concept for extracting quantitative association rules from numeric microarray datasets without using discretization. The limitation to this approach is that it was unable to find the complete set of significant rules from the microarray data. The GenMiner technique was proposed by Martinez et al. (2008) for finding association rules from a set of gene expression data and the online available terms that were linked to Gene Ontology (i.e., GO-terms). Bhadra et al. (2017), Mallik et al. (2013), and Bhadra et al. (2018) proposed a new concept where the cutoff (threshold) value was considered dynamical and altered for each gene set according to the quality/importance of the whole gene set rather than the quantification property. Some latest works are also based on the ARM/optimization method. Theilhaber et al. (2020) provided a tool in two-arm clinical studies. The methodology was based on the construction and optimization of a predictive multivariate gene signature that can predict the differential survival of patients undergoing anti-cancer therapies. Theilhaber et al. (2020) applied enhanced binary particle swarm optimization (EBPSO) in clinical transcriptomic cohorts to identify accurate, crisp, and significantly prognostic unique candidate signatures. The gene regulator within this signature yields biological insights into the relevant functions that were strongly correlated with their cancer type (Murphy et al., 2022). Nivedhitha et al. (2020) conducted survey research by categorizing different feature selection algorithms under supervised, unsupervised, and semi-supervised learning. This survey presented some latest tools of dimensionality reduction for tumor detection and also analyzed their performances and highlighted limitations and direction of future research to handle the high-dimension and less sample size data. On 2020, Ganguly and Mukherjee (2020) provided a modeling, simulation, and performance analysis study for an isolated hybrid power system (IHPS) which contained the solar thermal power plant, diesel engine generator (DEG), and wind turbine generator (WTG). To achieve better results for the studied IHPS model, authors applied the quasi-oppositional-based whale optimization algorithm and obtained better controller gain than other benchmark algorithms. In addition, there are some existing works of association rule mining which are based on fuzzy or rough theory (Sharmila and Vijayarani, 2019; Singh and Ganesh Wayal, 2012). However, the outcome rules are not good enough. Inclusion of the multi-objective optimization technique is an efficient step to improving the performance of association rule mining. After surveying the literature, we obtained some recently developed multi-objective optimization techniques that were presented by Mudi et al. (2019), Mudi et al. (2021a), Mudi et al. (2021b), Mudi et al., (2022), Ganguly et al. (2018), Ganguly et al. (2017), and Ganguly and Mukherjee (2020). In this article, we developed multi-objective optimized variable cutoff-based association rule mining (*MOOVARM*) for multi-omics profiles based on the minimum distance from the positive ideal solution (*PIS*) and that from the negative ideal solution (*NIS*). In this regard, we first identified (*PIS*) and (*NIS*) with respect to all gene sets. Therefore, we calculated the distance (*d* +) from *PIS* and distance (*d* −) from *NIS* for each product/item set. According to our proposed method, we calculated the relative closeness score value based on those two distances *d* + and *d* − for ranking the gene sets. If the relative closeness score of any rule was greater than or equal to the pre-defined cutoff value, the rule could be considered the final resultant rule. The proposed method calculated the relative closeness score globally instead of individual genes. Last, we made the ranking of the rules based on the relative score which had better disease classification performance than the state-of-the-art algorithms in disease diagnosis and therapeutic response.

## 2 Shortest distance-based cutoffs

The distance-based variable supports (denoted by *D*_*b*_ *VS*) cutoff technique proposed by Mallik and Zhao (2017b) was introduced to obtain some attractive rules from multi-omics datasets by combining co-expression, co-methylation, and protein–protein interactions. The normalized combined correlation score was calculated by the integration of co-expression and co-methylation values (say *CECM*_*exm*_) between the expression and methylation profiles containing a specific number of genes which are both differentially expressed and methylated. Basically, *CECM*_*exm*_ measures the similarity of expression and methylation patterns between the two genes. The expression/methylation data of all the diseased and control values are denoted by a gene vector *G*. Let *p* and *q* be two genes, and *CECM*_*exm*_ between *p* and *q* is denoted by *CECM*_*exm*_ (*p*, *q*). This is computed as follows:

$$CECM_{\mathrm{exm}}\left(p,q\right)=norm\left(PCB\left(G_{ex}\left(p\right),G_{ex}\left(q\right)\right)\;*\;r\left(G_{m}\left(p\right),G_{m}\left(q\right)\right)\right),$$ (1)

where *G*_*ex*_(*p*) and *G*_*m*_(*p*) are two vectors consisting of expression and methylation values, respectively, across all samples for the *p*th gene. Pearson’s correlation coefficient (Mallik, 2013) between the two groups is denoted by *r* (⋅, ⋅), where *PCB*(⋅, ⋅) processes the multiplication of Pearson’s correlation score and the BioSIM score (Bandyopadhyay and Bhattacharyya, 2011) between any two genes. Here, the normalization technique is denoted by *norm* (⋅) which followed the min–max normalization concept. The lower and upper limits *CECM*_*exm*_ (⋅, ⋅) were 0 and 1, respectively. Thereafter, the corresponding dissimilarity scores (say *D*_*simt*_) were computed with the help of *CECM*_*exm*_ scores, i.e., *D*_*simt*_ (*p*, *q*) = (1 − *CECM*_*exm*_ (*p*, *q*)). Thereafter, we determined protein–protein interactions from the Human Protein Resource Database (*HPRD*) and selected the interactions of the interactive protein-oriented genes among the set of genes which are differentially expressed and methylated. *H* is the protein–protein interaction matrix for the selected differentially expressed and methylated genes. In every gene pair (*p*, *q*), we multiplied the interaction value in *H* and the corresponding weighted distance value in *D*_*simt*_ and subsequently calculated the resultant value, *DijStP* (*p*, *q*). The expression of *DijStP* (*p*, *q*) is given as *DijStP* (*p*, *q*) = (*H* (*p*, *q*)*D_*simt*_ (*p*, *q*)). To compute DijStP (p,q) for all gene pairs (*p*, *q*), we selected the weighted distance for every gene pair that contained the interactions in their corresponding protein levels among each other. This resulted in a similarity and symmetric matrix. Using this matrix, we constructed a weighted transcriptomic gene regulatory network. Dijkstra’s shortest path algorithm was then used on the gene regulatory network, and the relative weighted shortest distance matrix was generated (denoted by *W*_*e*_ SD). According to the fundamental biological theory, the biological functions or biological pathways of two genes are the same if the distance between two genes is low. In this work, we utilized the shortest distance between every two genes belonging to the network. Thereafter, we calculated different distances among all gene pairs belonging to the *W*_*e*_ SD matrix such as the maximum weighted shortest distance (*W*_*e*_ SD_*mx*_), minimum weighted shortest distance (*W*_*e*_ SD_*mn*_), and average weighted shortest distance (*W*_*e*_ SD_*avg*_) that were computed without considering the diagonal elements of the underlying matrix. The distance-based variable supports threshold within the gene set (*GS*) *D*_*b*_ *VS*(*GS*) is defined as follows:

$$D_{b}VS\left(GS\right)=\sqrt{\frac{1}{ngp}\underset{p,q\in GS;p\neq q}{\sum }WV_{\mathrm{msc}}{\left(p,q\right)}^{2}},$$ (2)

where

$$WV_{\mathrm{msc}}\left(p,q\right)=\left\{\begin{array}{cc} UVminS\left(1-\frac{\left(WeSD\left(p,q\right)-med\left(WeSD\right)\right)*c1}{c2*MAD\left(WeSD\right)}\right),\; & \text{ if}p!=q,\\ UVminS,\; & \text{ if}p==q, \end{array}\right.$$

where *ngp* indicates the total number of possible gene pairs within *GS* and *c*1 and *c*2 are two constant terms. The value for *c*1 is set at 0.10, while *c*2 is a constant scaling factor whose value is set at 1.4826 for the assumption of a Gaussian distribution pattern to utilize any parametric test.

Similarly, another two different types of thresholds, *viz.*, distance-oriented variable confidence (denoted by *D*_*b*_ *VC*) and distance-oriented variable lift (denoted by *D*_*b*_ *VL*), are defined as follows:

$$D_{b}VC\left(GS\right)=\sqrt{\frac{1}{ngp}\underset{p,q\in GS;p\neq q}{\sum }WV_{\mathrm{mcc}}{\left(p,q\right)}^{2}},$$ (3)

where

$$WV_{\mathrm{mcc}}\left(p,q\right)=\left\{\begin{array}{cc} UVminC\left(1-\frac{\left(WeSD\left(p,q\right)-med\left(WeSD\right)\right)*c1}{c2*MAD\left(WeSD\right)}\right),\; & \text{ if}p!=q,\\ UVminC,\; & \text{ if}p==q, \end{array}\right.$$

where *UVminC* depicts the user-mentioned minimum confidence threshold, and

$$D_{b}VL\left(GS\right)=\sqrt{\frac{1}{ngp}\underset{p,q\in GS;p\neq q}{\sum }WV_{\mathrm{mlc}}{\left(p,q\right)}^{2}},$$ (4)

where

$$WV_{\mathrm{mlc}}\left(p,q\right)=\left\{\begin{array}{cc} UDminL\left(1-\frac{\left(WeSD\left(p,q\right)-med\left(WeSD\right)\right)*c1}{c2*MAD\left(WeSD\right)}\right),\; & \text{ if}p!=q,\\ UDminL,\; & \text{ if}p==q, \end{array}\right.$$

where *UDminL* represents the user-mentioned minimum lift threshold value, while *c*1 and *c*2 denote the constant values for scaling the fractional part.

## 3 Multi-objective optimized association rule mining for the multi-omics dataset

In this section, we developed a novel algorithm called *MOOVARM* for multi-omics profiles. Here, we integrated the gene expression, methylation, and protein–protein interaction data based on the idea of multi-objective optimization and weighted shortest distance to produce interesting rules for multi-omics profiles. The three basic steps of this algorithm are explained in the following sections. All abbreviations of Model parameters are discussed in Table 1.

**TABLE 1:** Model parameters.

Symbol	Definition
*CECM*_*exm*_	Co-expression and co-methylation values
*G*	Gene vector
norm	Normalization technique
PCB	Pearson’s correlation coefficient
*G*_*ex*_	Vectors consisting of the expression and methylation values
*D*_*simt*_	Dissimilarity scores
*HPRD*	Human Protein Resource Database
*DijStP* (*p*, *q*)	The weighted distance for every gene-pair (p, q) that contained the interactions in their corresponding protein levels among each other
*W*_*e*_ SD	Weighted shortest distance matrix
*W*_*e*_ SD_*mx*_	Maximum weighted shortest distance
*W*_*e*_ SD_*mn*_	Minimum weighted shortest distance
*W*_*e*_ SD_*avg*_	Average weighted shortest distance
*WV*_*msc*_	Minimum support threshold
*WV*_*mcc*_	Minimum confidence threshold
*WV*_*mlc*_	Minimum lift threshold
*UDminS*	User-defined minimum support threshold
*UD*_min_ *C*	User-mentioned minimum confidence cutoff
*UD*_min_ *L*	User-defined minimum lift cutoff
med	Median value
MAD	Median absolute deviation
*GS*	Gene set
*GSTR*	Gene set tree
*ngp*	Total number of possible gene pairs
*D*_*b*_ *VS*	Distance-based variable supports threshold
*D*_*b*_ *VC*	Distance-oriented variable confidence
*D*_*b*_ *VL*	Distance-oriented variable lift
*DiEM*_norm_	Set of normalized genes
*DiE*_norm_	Normalized expression
*DiM*_norm_	Normalized methylation data matrices
*UpR*	Upregulated
*DwR*	Downregulated
*HpoM*	Hypo-methylated
*DDiE*_norm_	Discretized expression
*DDiM*_norm_	Methylation data matrices
*DDiTE*_norm_	Transposed discretized expression data
*DDiTM*_norm_	Methylation data
*PDiD*_*em*_	Binary matrix
*TRDiM*	Transactional matrix
*TR*_*n*_	Number of transactions
*M*_*nm*_	Decision matrix
*p*_*ij*_	Choice value
*PIS* ^+^	Positive ideal solution
*NIS* ^−^	Negative ideal solution

### 3.1 Finding significant genes

Initially, matched genes and matched samples between gene expression and methylation data were found. Using the zero-mean normalization (Bandyopadhyay et al., 2014) technique, the gene expression/methylation data were normalized genewise. The empirical Bayes test using the limma package (Mallik and Zhao, 2017b; Mallik and Zhao, 2017a; Smyth, 2004) on both normalized expression and methylation data was executed for finding differentially expressed and methylated genes. limma was used because of its effectiveness on normalized gene expression/methylation data for any data distribution and any number of samples. Numerous pairs of genes in the normalized expression/methylation dataset comprised more than one probe. We applied limma for every gene probe individually and found the differentially expressed/methylated probes in terms of the significant Benjamini–Hochberg (BH) corrected *p*-value. The probes for which the Benjamini–Hochberg (BH) corrected *p*-value is less than the standard cutoff 0.05, the expression/methylation data are treated as differentially expressed/methylated gene probes. Then, we selected the probe of each gene for which the corresponding Benjamini–Hochberg (BH) corrected *p*-value generated using the limma tool was the lowest among all probes of each gene. The remaining probes of those genes were deducted from the corresponding dataset. Last, only those genes containing single probes were obtained which were both differentially expressed and methylated and whose respective proteins had interactions in the *HPRD*.

### 3.2 Discretization and post-discretization formats

Assuming that *N* referred to the set of genes which had both the differential expression and differential methylation profiles and which were involved in the protein–protein interaction, while *n* denotes the number of genes that are both differentially expressed and differentially methylated (*N*). Let *M* denote the set of matched samples between the expression and methylation data, while *m* denotes the number of matched samples between the expression and methylation data (*M*). The normalized expression and methylation data matrices of the genes belonging to *N* are symbolized as *DiE*_norm_ and *DiM*_norm_, respectively. The row of data matrices represents the gene, whereas the column indicates the transaction (sample). The binary representation of *DiE*_norm_ and *DiM*_norm_ is essential for the association rule mining. When *DiE*_norm_ was normalized applying the zero-mean normalization technique, the rowwise (i.e., genewise) mean values became zero. If the value of expression data was greater than 0, the value was treated as upregulation (denoted as *UpR*), and thus, it was converted into 1 at the time of discretization, whereas any value which was less than 0 denoting downregulation (denoted by *DwR*) was turned into 0 during discretization. On the other hand, in the methylation data, the value that was greater than 0 indicating hyper-methylation (denoted as *HperM*) was converted into 0, whereas any value that was less than 0 indicating hypo-methylation (denoted as *HpoM*) was converted into 1. The aforementioned discretization procedure for the expression and methylation datasets is described in the following equations, respectively:

$$DDiE_{\mathrm{norm}}\left(i,j\right)=\left\{\begin{array}{cc} 1,\; & \text{ if }DiE_{\mathrm{norm}}\left(i,j\right)>0,\\ \text{0},\; & \text{ if }DiE_{\mathrm{norm}}\left(i,j\right)<0, \end{array}\right.$$ (5)

$$DDiM_{\mathrm{norm}}\left(i,j\right)=\left\{\begin{array}{cc} 1,\; & \text{ if }DiM_{\mathrm{norm}}\left(i,j\right)<0,\\ \text{0},\; & \text{ if }DiM_{\mathrm{norm}}\left(i,j\right)>0, \end{array}\right.$$ (6)

where *DDiE*_norm_ and *DDiM*_norm_ indicate the discretized expression and methylation data matrices, respectively. The range of *i* and *j* values are 1–*n* and 1–*m*, respectively. Then, all the resultant discretized matrices are transposed as follows:

$$DDiTE_{\mathrm{norm}}=t\left(DDiE_{\mathrm{norm}}\right),$$ (7)

and

$$DDiTM_{\mathrm{norm}}=t\left(DDiM_{\mathrm{norm}}\right).$$ (8)

During post-discretization, the transposed discretized expression data (denoted by *DDiTE*_norm_ in Eq. 7) and methylation data (denoted by *DDiTM*_norm_ in Eq. 8) were merged into a single binary matrix (denoted by *PDiD*_*em*_), with the size of [*m* × (2*n)]. The integration of the expression and methylation data produced four types of genes, *viz.*, 1) upregulated and hypo-methylated genes, 2) upregulated and hyper-methylated genes, 3) downregulated and hyper-methylated genes, and 4) downregulated and hypo-methylated genes. As gene expression and methylation are inversely proportional to each other, the first and third categories of gene sets (i.e., categories denoted by (i) and (iii)) were selected. As mentioned previously, the column length (gene area) of post-discretization is twice that of the column length (gene area) of the transposed discretized expression/methylation matrix, i.e., the size of *PDiD*_*em*_ is [*m* × (2*n)].

The first half of the column vector of *PDiD*_*em*_ is for type (i) upregulation and hypo-methylation, while the second half of the column vector of *PDiD*_*em*_ is for type (iii) downregulation and hyper-methylation. Therefore, if the particular cell/house value (say cell at the jth sample and the ith gene) of the transposed discretized expression data matrix *DDiTE*_norm_ is 1 (i.e., the so-called upregulated) and the same cell/house value of the transposed discretized methylation data matrix *DDiTM*_norm_ is 1 (i.e., the so-called hypo-methylation), it satisfies type (i) upregulation and hypo-methylation. We place a symbol “1” at the same cell/location of the first half of the post-discretized matrix (i.e., cell at the jth sample and the ith gene of *PDiD*_*em*_ that are seen as the first joint condition of Eq. 9) that indicated type (i) both upregulated and hypo-methylated genes, and simultaneously we also place a symbol “0” at the same cell/location of the second half of the post-discretized matrix (i.e., cell at the jth sample and the ith gene of *PDiD*_*em*_ that are seen as the first joint condition of Eq. 10) which is just the negation of “1.” On the other hand, when both the transposed discretized scores for the same cell/house were 0 (downregulation and hyper-methylation), the resultant post-discretized value for the second half of the post-discretized matrix would be 1 (see the second joint condition of Eq. 10), whereas the same value for the first half of the post-discretized matrix would automatically be the negation of 1 (*viz.*, 0) (see the second joint condition of Eq. 9). In addition, for all the other combinations of the transposed discretized expression value and the transposed discretized methylation value [e.g., (0 and 1), (1 and 0)], the post-discretized values for both the first and second half would be 0.

$$PDiD_{em}\left(j,i\right)=\left\{\begin{array}{cc} 1,\; & \text{ if }DDiTE_{\mathrm{norm}}\left(j,i\right)==1\&DDiTM_{\mathrm{norm}}\left(j,i\right)==1,\left(UpR\;\&\;HypoM\right),\\ \text{0},\; & \text{ if }DDiTE_{\mathrm{norm}}\left(j,i\right)==0\&DDiTM_{\mathrm{norm}}\left(j,i\right)==0,\left(DwR\;\&\;HyperM\right),\\ \text{0},\; & \text{ otherwise}, \end{array}\right.$$ (9)

and

$${\text{PDiD}}_{em}\left(j,i+n\right)=\left\{\begin{array}{cc} \text{0},\; & \text{ if }DDiTE_{\mathrm{norm}}\left(j,i\right)==1\&DDiTM_{\mathrm{norm}}\left(j,i\right)==1,\\ 1,\; & \text{ if }DDiTE_{\mathrm{norm}}\left(j,i\right)==0\&DDiTM_{\mathrm{norm}}\left(j,i\right)==0,\\ \text{0},\; & \text{ otherwise}. \end{array}\right.$$ (10)

**FIGURE 1:**
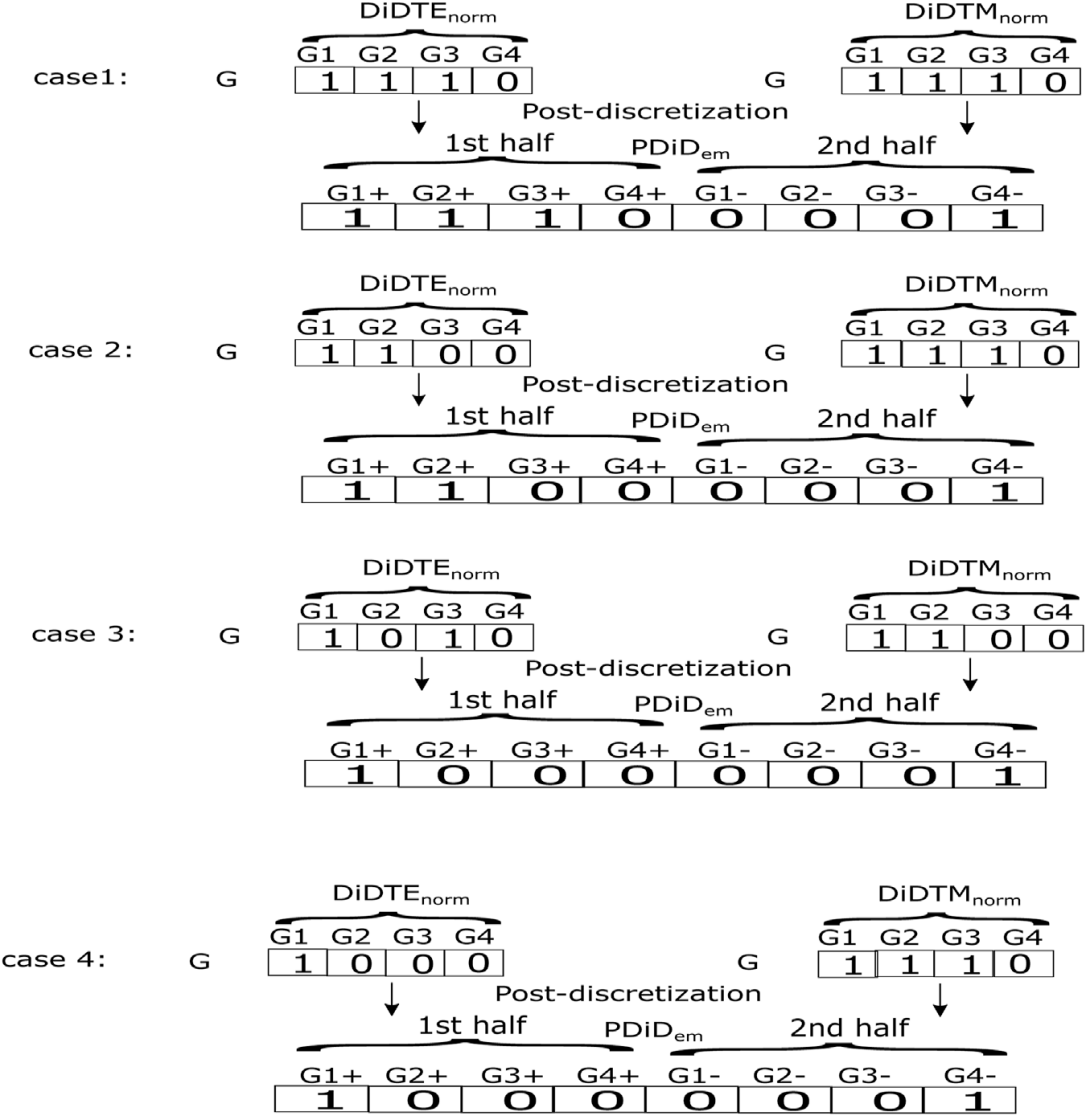
Examples of post-discretization in the proposed method.

However, we illustrated some examples of aforementioned computations in Figure 1. After the post-discretization step, we carried out transpose on the resultant post-discretized matrix for the next step.

### 3.3 Proposed association rule mining approach

The *MOOVARM* approach changed the traditional concept of using the static support threshold and a static confidence threshold which were generally applied to maintain these same thresholds across all item sets (i.e., gene sets). In our method, after post-discretization, the association rule mining algorithm utilized the weighted shortest distance depending on multiple minimum support thresholds, multiple minimum confidence thresholds, and multiple minimum lift thresholds instead of the static support threshold and the static confidence threshold. Those multiple minimum thresholds were formed through the integration of gene expression, methylation, and protein–protein interaction profiles. The *MOOVARM* method worked on three different types of profiles: gene expression, methylation, and protein–protein interaction profiles concurrently instead of the individual dataset, like gene expression or DNA methylation or any other data, and produced multi-objective multi-prolific association rules. The six main steps of this *MOOVARM* method were as follows: 1) determination of frequency of every gene (item) contained in the post-discretized data; 2) computation of *WV*_*msc*_, *WV*_*mcc*_, and *WV*_*mlc*_ scores; 3) formation of a gene set tree (*GSTR*); 4) generation of gene sets (item sets); 5) determination of *D*_*b*_ *VS*, *D*_*b*_ *VC*, and *D*_*b*_ *VL*; and 6) production of the top significant relation-dependent association rules.

In the first step, the binary matrix denoted by *PDiD*_*em*_ was transformed into the transactional matrix *TRDiM*, which contained transactions associated with several genes IDs per transaction. The number of transactions that existed in *TRDiM* was denoted by *TR*_*n*_. Both the user-mentioned minimum support cutoff (*UD*_min_ *S*) and user-mentioned minimum confidence cutoff (*UD*_min_ *C*) were to be described. *UD*_min_ *L* (user-defined minimum lift cutoff) was kept at the value 1. Then, the frequency of every gene from the *TRDiM* dataset was determined. The frequency of the genes was greater than or equal to *UD*_min_ *S* and were considered frequent genes. The frequent genes were arranged according to their frequency (from high to low order). In the second step, the generated cutoff *WV*_*msc*_ (;) was computed for every pair of genes by combining *H* (;), *CECM*_*exm*_ (;), and *UD*_min_ *S*. Similarly, *WV*_*mcc*_ (;) was evaluated by applying *H* (;), *CECM*_*exm*_ (;), and *UD*_min_ *C*, and *WV*_*mlc*_ (;) was computed by integrating *H* (;), *CECM*_*exm*_ (;), and *UD*_min_ *L*. In the next two phases, the *GSTR* was first obtained, and the important gene sets were then generated consecutively by following the same steps used in the typical *FP*-*Growth* association rule mining method. Next, in the fifth phase, the distance-based cutoff (i.e., *D*_*b*_ *VS*, *D*_*b*_ *VC*, and *D*_*b*_ *VL*) scores were evaluated for every resultant gene set by the initially computed *WV*_*msc*_, *WV*_*mcc*_, and *WV*_*mlc*_ matrices, successively. In the final phase, the support of every resultant gene set was first identified. The frequent gene sets (i.e., the gene sets whose support scores were greater than or equal to the respective individual *D*_*b*_ *VS* threshold instead of the user-specified support threshold *UD*_min_ *S*) were then identified. Next, the rules were obtained with respect to the frequent gene sets, and the confidences and lifts of the respective rules were computed. From the aforementioned set of rules, we chose only those rules for which both the confidence and lift scores were greater than or equal to their individual *D*_*b*_ *VC* and *D*_*b*_ *VL* cutoffs instead of *UD*_min_ *C* and *UD*_min_ *L*, respectively.

**FIGURE 2:**
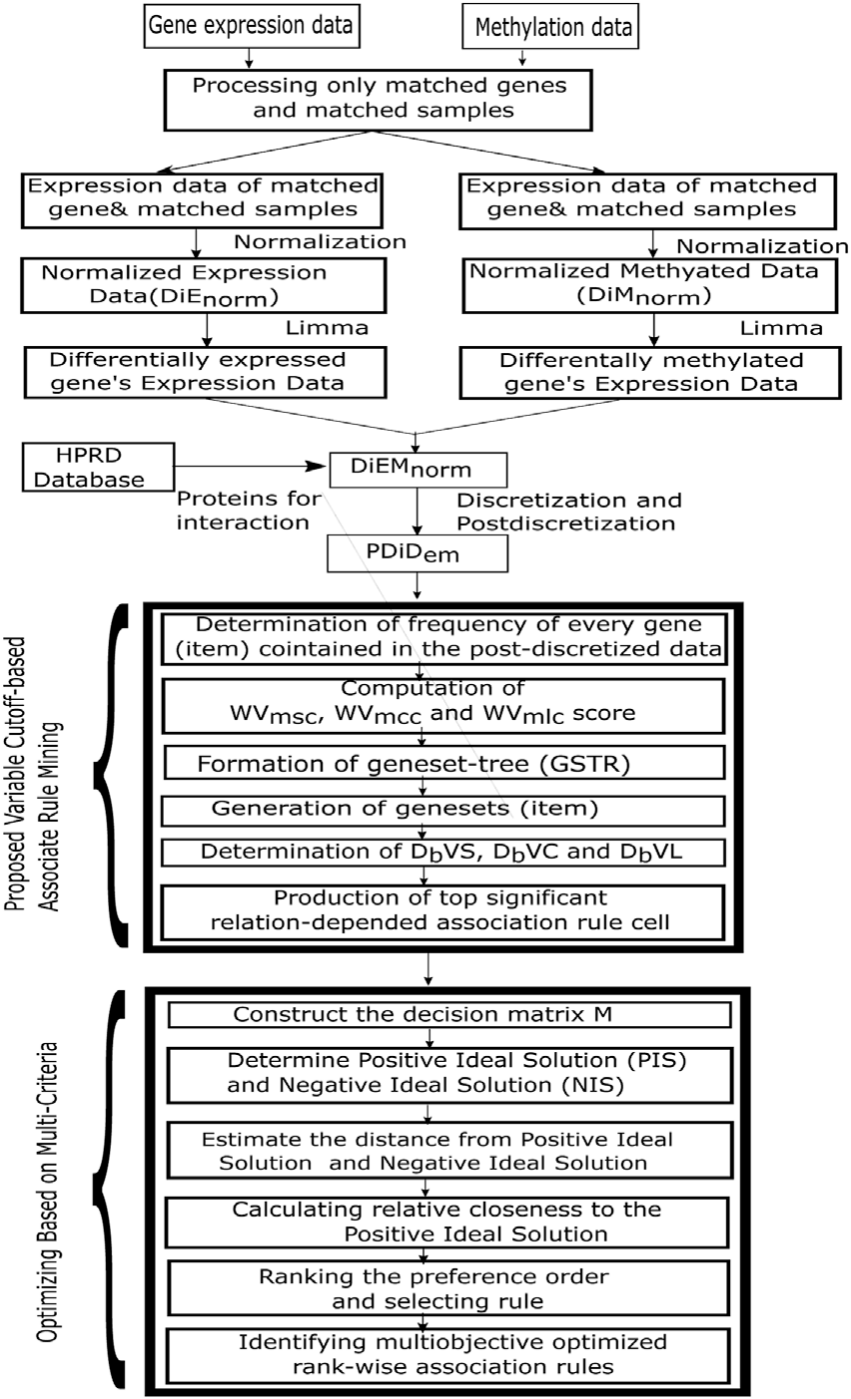
Flowchart of the proposed rule mining method.

A flowchart of the proposed *MOOVARM* rule mining method is illustrated in Figure 2.

## 4 Multi-criteria (multi-objective optimization) decision-making technique

Multi-criteria decision-making (*MCDM*) (Das et al., 2013) is a procedure used to select the best alternative of the set of finite alternatives with respect to multiple criteria. The *MCDM* technique has various applications in different fields such as economy, management, engineering, and medical diagnosis and helps the decision maker in selecting the best alternative in conflicting situations.

Algorithm 1. *MOOVARM*

**Input:** Gene expression $\left(EX\right)$ , DNA methylation data $\left(Mt\right)$ and protein–protein interaction $\left(PPI\right)$ data
**Output:** List of rank wise multi objective optimized association rules
1:  Procedure MOOVARM(*EX*, *Mt*, *PPI*)
2:   Find the matched genes and matched samples between *EX* and *Mt*, and choose only them for *EX*/*Mt*
3:   Normalize *EX*/*Mt* by zero-mean normalization
4:   Identify differentially expressed genes from *EX* and differentially methylated genes from *Mt*; and intersect them and finally choose those intersected genes that have interactions in *HPRD* (denoted as *DiEM*_norm_ gene set)
5:   Discretize the *EX*/*Mt* subdata having *DiEM*_norm_ gene set into *DDiE*_norm_ (:) and *DDiM*_norm_ (:), respectively, and post-discretize them together into a single matrix, *PDiD*_*em*_. (See Eqs 5–10)
6:   Transpose *PDiD*_*em*_ into the transactional matrix *TRDiM*
7:   Generate frequent gene set *GS* from *TRDiM*
8:   **for** each gene *g*_*i*_ ∈ *TRDiM* **do**
9:    **if** frequency (*g*_*i*_) ≥ *UD*_min_ *S* **then**
10:     *GS* ← *g*_*i*_
11:    **end if**
12:   **end for**
13:   Determine *WV*_*msc*_, *WV*_*mcc*_, and *WV*_*mlc*_ cutoff scores for each pair of gene
14:   Form gene set tree (*GSTR*) and then generate important gene set by FP-Growth rule mining method
15:   Distance-based cutoff (i.e., *D*_*b*_ *VS*, *D*_*b*_ *VC*, *D*_*b*_ *VL*) scores were evaluated for every resultant gene set using *WV*_*msc*_ (:), *WV*_*mcc*_ (:), and *WV*_*mlc*_ (:) scores, successively. Produce top significant relation-dependent association rules. (See Eqs 2–4)
16:   Develop the decision matrix *M* according to Confidence, Support, Lift, and Average WeSD value of rules
17:   Determine the Positive Ideal Solution (*PIS* ^+^) and Negative Ideal Solution (*NIS* ^−^) (See Eqs 11 and 12)
18:   Calculate the distance ( $DIS_{i}^{+}$ ) using *PIS* ^+^ and the distance ( $DIS_{i}^{-}$ ) from *NIS* ^−^ of each alternatives (See Eqs 13 and 14)
19:   Compute the relative closeness (*S*_*i*_) to the positive ideal solution of each alternative (See Eq. 15)
20:   Ranking the preference order according to relative closeness and select the alternative that is close to 1. Thereafter, rank the alternative depending on *S*_*i*_ score in descending order
21:  **end procedure**

**FIGURE 3:**
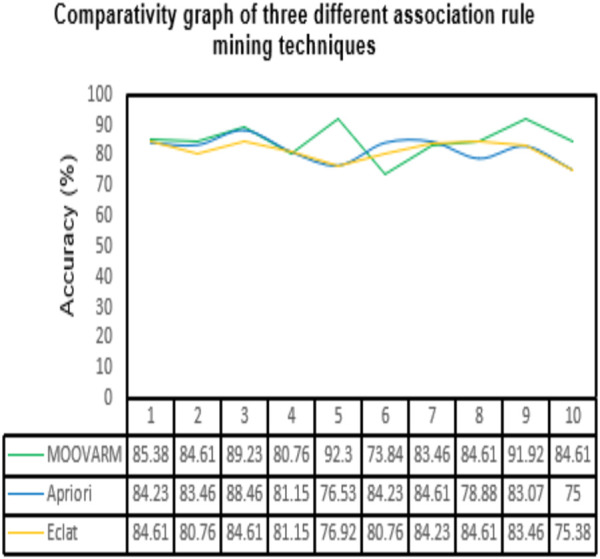
Comparative study between our proposed method *MOOVARM* and other well-known related rule mining methods, *Apriori* and *Eclat*, in terms of classification accuracy of the generated top 10 rules obtained by **(A)** *MOOVARM*, **(B)** *Apriori*, and **(C)** *Eclat*.

During the decision-making process, the decision-maker considers the number of criteria which is helpful in reaching the goal. Among those criteria, some conflict with each other, some are maximized, and some are minimized. Those types of problems are solved by different *MCDM* techniques such as *MAXMIN* (Shen and Mo, 2009), *MAXMAX* (Shen and Mo, 2009), *AHP* (Saaty, 1990), *ELECTRE* (Roy and Vanderpooten, 1996), and *TOPSIS* (Hwang and Yoon, 1981). Those methods are considered a decision-making procedure depending on the problem behaviors such as ranking, scoring, selecting, ordering, and surrounding environments such as available data type and size, processing/execution time, internal consistency, and logical relations. The *TOPSIS* method is the most suitable *MCDM* method under two cases: first, in case of problems related to the large number of criteria and alternatives; second, in case of availability of objective and quantity data. First, the *TOPSIS* method identifies the positive ideal alternative which has the extreme performance on each criterion. It also identifies the negative ideal alternative that produced the worst performance on each criterion. The positive ideal solution is the solution that maximizes the benefit criterion and minimizes the cost criterion, whereas the negative ideal solution maximizes the cost criterion and minimizes the benefit criterion. Next, the method finds the alternative, depending on the closest distance from the positive ideal solution and farthest distance from the negative ideal solution. The classical *TOPSIS* method was based on the information of the criteria that was collected from the expert opinions and quantitative data, whereas the generated solution was concentrated on evaluation, prioritization, and selection (Figure 3).

The TOPSIS method calculates relative closeness and ranking through the following steps.

**Step 1:** Constructing the decision matrix.Let $M={\left(p_{ij}\right)}_{\mathrm{nxm}}$ correspond to a decision matrix, where *p*_*ij*_ indicates the choice value of the *i*th alternative and *j*th criteria.

**Step 2:** Determining the positive ideal solution (*PIS* ^+^) and negative ideal solution (*NIS* ^−^). The positive ideal solution (*PIS* ^+^) is denoted as follows:

$$\begin{array}{c} PIS^{+}=\left\{p_{1}^{+},p_{2}^{+},\ldots \ldots .p_{m}^{+}\right\}=\\ \left\{\left({max}_{i}\left(p_{ij}\right)|j\in K\right),\left({min}_{i}\left(p_{ij}\right)|j\in L\right)\right\}. \end{array}$$ (11)

The negative ideal solution (*NIS* ^−^) is denoted as follows:

$$\begin{array}{c} NIS^{-}=\left\{p_{1}^{-},p_{2}^{-},\ldots \ldots .p_{m}^{-}\right\}=\\ \left\{\left({min}_{i}\left(p_{ij}\right)|j\in K\right),\left({max}_{i}\left(p_{ij}\right)|j\in L\right)\right\}, \end{array}$$ (12)

where K is associated with the benefit criteria and L is associated with the cost criteria.

**Step 3:** Calculating the distance from the positive ideal solution and negative ideal solution. The distance of the *i*th alternative from the positive ideal solution $DIS_{i}^{+}$ is then calculated accordingly as follows:

$$DIS_{i}^{+}=\left(\overset{m}{\underset{j=1}{\sum }}\left(p_{j}^{+}-p_{ij}\right)\right),\;i=1,2,\ldots \ldots n,$$ (13)

while the distance of the *i*th alternative from the negative ideal solution $DIS_{i}^{-}$ is then computed as follows:

$$DIS_{i}^{-}=\left(\overset{m}{\underset{j=1}{\sum }}\left(p_{ij}-p_{j}^{-}\right)\right),\;i=1,2,\ldots \ldots n.$$ (14)

**Step 4:** Calculating the relative closeness to the positive ideal solution *S*_*i*_ as follows:

$$S_{i}=\frac{DIS_{i}^{+}}{DIS_{i}^{+}+DIS_{i}^{-}},where\;\;0<S_{i}<1,\;i=1,2,\ldots \ldots n.$$ (15)

**Step 5:** Ranking the preference order, and selecting the alternative close to 1. Ranking of the alternatives depending on the *S*_*i*_ score was made in descending order.

**FIGURE 4:**
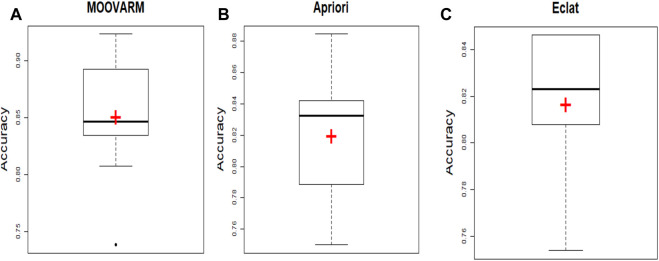
Comparative study between our proposed method *MOOVARM* and other well-known related rule mining methods, *Apriori* and *Eclat*, in terms of mean classification accuracy of the generated rules obtained by **(A)** *MOOVARM*, **(B)** *Apriori*, and **(C)** *Eclat*, where “+” (red symbol) denotes the average classification accuracies and the bold line signifies their median.

Notably, see Algorithm 1 for the major steps of the proposed algorithm MOOVARM (Figure 4).

## 5 Experimental datasets and results

In the experiment, integrative data consisting of DNA methylation and gene expression high-grade soft tissue sarcoma (*HSTS*) profiles (NCBI ID: GSE52392) (Renner et al., 2013; Chudasama et al., 2017) were utilized. At the initial stage, the methylation profile had 27,578 methylation probes, whereas the gene expression profile consisted of a total of 48,645 genes. Of note, we selected those samples which contained both the values that consisted of two categories of samples: (i) undifferentiated pleomorphic liposarcoma (*UdPLs*) (diseased samples) and (ii) normal tumor cell line (*nrTCL*) (i.e., control samples). The profile had 13 *UdPLs* samples and 13 *nrTCL* samples. Thereafter, we chose the matched genes (i.e., 12,438) that consisted of both methylation and expression values.

**FIGURE 5:**
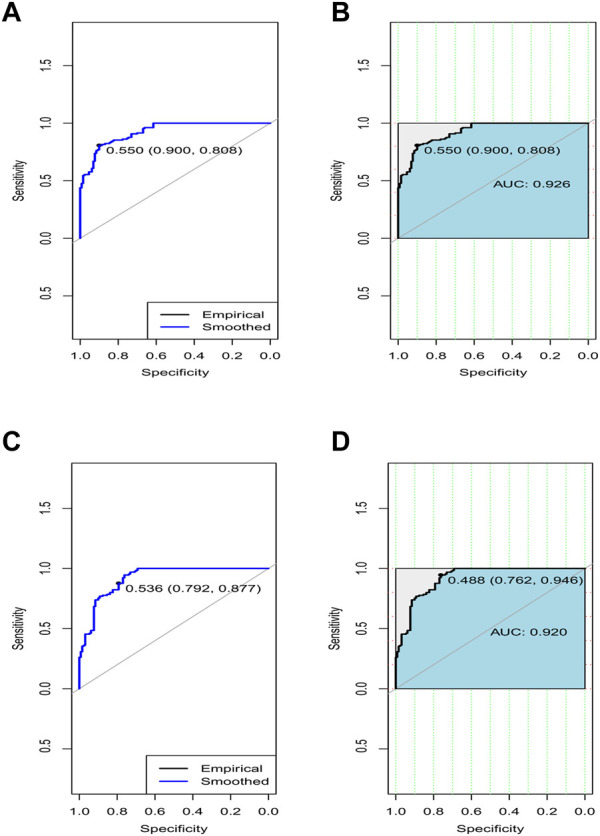
ROC curves of average accuracies and AUC in terms of sensitivity vs. specificity to classify the respective disease using the gene sets belonging to the topmost two rules by MOOVARM (of Table 3) from the respective dataset. **(A)** ROC curve of avg. accuracies to classify the respective disease using the gene sets belonging to the first rule, **(B)** AUC to classify the respective disease using the gene sets belonging to the first rule, **(C)** ROC curve of avg. accuracies to classify the respective disease using the gene sets belonging to the second rule, and **(D)** AUC to classify the respective disease using the gene sets belonging to the second rule. Of note, the blue curve denotes the empirical line, while the light black line indicates the smoothed control (X:Y = 1:1) line. Herein, “0.550 (0.900, 0.808)” denotes the optimal threshold point (=0.550) that is closest to the top left part of the plot (for rule 1), where the respective specificity and sensitivity are 0.900 and 0.808, respectively. Similarly, “0.536 (0.792, 0.877)” signifies the optimal threshold point (=0.536) that is closest to the top left part of the plot (for rule 2), where the respective specificity and sensitivity are 0.792 and 0.877, respectively.

During the experiment, we first selected the genes that contained both DNA methylation and expression values. Since genes had more than one single probe for methylation and expression profiles, we preliminarily filtered out those probes containing the missing values. The limma R tool (Smyth, 2004) was then applied on each probe to know whether the probe was differentially expressed/methylated or not (Figure 5).

The probe with the best significance (minimal corrected *p*-value) among all the probes for every gene was selected for the next analysis, whereas all the remaining probes for every gene were simply omitted from the methylation profile and the expression profile. Next, we conducted the intersection between the set of differentially methylated genes, the set of differentially expressed genes, and the set of genes whose respective proteins interacted with one another in the *HPRD* (Peri et al., 2003). Herein, we identified many such common genes. For each dataset, we constructed a protein–protein interaction (*PPI*) network where each protein denoted a gene in the respective intersected set of genes. Next, we calculated the degree of each node (gene) in the *PPI* network and rearranged the genes with respect to the high to low order of their degree values. Thereafter, we conducted the discretization and post-discretization steps, respectively. Then, we used our proposed rule mining method, *MOOVARM*, and obtained multi-objective optimized variable support-based association rule mining. Table 2 shows the resultant rules. Notably, using the four measures (confidence, support, lift, and *WeSD*) of each rule, we optimized the rules through computing the relative score in optimization where confidence, support, and lift were used to maximize their values and *WeSD* was used to minimize their values. Then, we ranked the rules according to the relative score from high to low. For the *HSTS* dataset, the topmost rule {STAT3+, TP53- → MAPK3+} states that if the gene *STAT*3 is both upregulated and hypo-methylated and the gene *TP*53 is both downregulated and hyper-methylated, then it is likely that the gene *MAPK*3 is upregulated and hypo-methylated. The confidence, support, lift, avg. *WeSD*, and relative score values of this rule are 0.01, 0.00269, 0.02275, 0.00543, and 0.36, respectively. Its previous rank before optimization was 4, but after optimization, it secured the first rank since it has the highest relative score among all the rules. The next top four ranked optimized rules are {STAT3+ → MAPK3+}, {JUN+, STAT3+, TP53- → MAPK3+}, {ESR1+ → MAPK3+}, and {JUN+, STAT3+ → MAPK3+}, whose relative scores are 0.3596, 0.3588, 0.3565, and 0.355, respectively (in Table 2). All details of the different rule interestingness measures, WeSD, relative scores, and the ranks prior to and after optimization for these top genes generated by MOOVARM are described in Table 2.

**TABLE 2:** Ranks of the evolved rules prior to optimization and after optimization in MOOVARM along with several rule interestingness measures, confidence, support, and lift, as well as average WeSD and relative scores.

***Rank***_***prevMOO***_	Rule	Confidence	Support	Lift	Avg. WeSD	Relative score	*Rank*_*afterMOO*_
1	STAT3+ → MAPK3+	0.009	0.003461538	0.0195	0.00339	0.3596	2
2	TP53- → MAPK3+	0.008888889	0.003076923	0.01925926	0.00408	0.3547	6
3	MAPK3+, TP53- → STAT3+	0.00875	0.002692308	0.02275	0.00543	0.346	15
4	STAT3+, TP53- → MAPK3+	0.01	0.002692308	0.02166667	0.003735	0.36	1
5	ESR1+ → MAPK3+	0.008333333	0.001923077	0.01805556	0.00357	0.3565	4
6	JUN+, STAT3+ → MAPK3+	0.008571429	0.002307692	0.01857143	0.00388	0.355	5
7	JUN+, FYN+ → MAPK3+	0.01	0.002307692	0.02166667	0.008	0.3354	22
8	STAT3+, FYN+ → MAPK3+	0.01	0.002692308	0.02166667	0.00751	0.3384	19
9	JUN+, TP53- → MAPK3+	0.008571429	0.002307692	0.01857143	0.004225	0.3525	7
10	JUN+, STAT3+, TP53 → MAPK3+	0.01	0.001923077	0.02166667	0.003946667	0.3588	3
11	FYN+, TP53 → MAPK3+	0.01	0.002307692	0.02166667	0.007855	0.3361	21
12	JUN+, AR+ → MAPK3+	0.01	0.001923077	0.02166667	0.00567	0.3474	14
13	JUN+, AR+ → TP53-	0.01	0.001923077	0.02888889	0.0072	0.3399	18
14	TP53-, AR+ → JUN+	0.01	0.001923077	0.026	0.00541	0.3494	13
15	JUN+, AR+ → MAPK3+, TP53-	0.01	0.001923077	0.0325	0.006435	0.3444	17
16	MAPK3+, JUN+, AR+ → TP53-	0.01	0.001923077	0.02888889	0.00616	0.3454	16
17	TP53-, AR+ → MAPK3+, JUN+	0.01	0.001923077	0.0325	0.0054675	0.3496	12
18	MAPK3+, TP53-, AR+ → JUN+	0.01	0.001923077	0.026	0.005063333	0.3514	9
19	JUN+, TP53-, AR+ → MAPK3+	0.01	0.001923077	0.02166667	0.00514	0.351	11
20	GRB2 → STAT3-	0.01	0.002692308	0.02888889	0.01145	0.324	23
21	FYN → TP53+	0.01	0.003076923	0.026	0.01186	0.3228	24
22	ESR1-, FYN → TP53+	0.01	0.002307692	0.026	0.00783	0.337	20
23	ESR1-, MAPK3 → STAT3-	0.01	0.001923077	0.02888889	0.005065	0.3516	8
24	STAT3-, MAPK3 → ESR1-	0.01	0.001923077	0.02888889	0.005155	0.3511	10

*“+” denotes upregulated and hypo-methylated genes; “−” represents downregulated and hyper-methylated genes.

In order to validate the significance of each of the top 10 rules (in Table 2) generated from *DTFP*-*Growth*, we used and executed the PAM classifier for comparing the classification performance of the different rules obtained from MOOVARM, Apriori, and Eclat rule mining methods toward the samples. For this purpose, we considered only the participating features (genes) from both sides of each individual rule of the top 10 rules and then ran 10-fold cross-validation on the data with the help of the PAM classifier with the default parameters to evaluate the importance of the combination of all genes participating in the rule. We repeated the entire procedure 10 times in every occasion. The obtained classification accuracies of the top 10 rules of the *HSTS* dataset are presented in Table 3. Similarly, accuracy values of the top 10 rules as per the *Apriori* and *Eclat* algorithms are displayed in Table 4 and Table 5, respectively. The graphical plot for the top 10 rules classification accuracy measures obtained by three methods, namely, *MOOVARM*, *Apriori*, and *Eclat* is presented in Figure 3. According to the top 10 classification accuracy metrics, it is clear that the overall accuracy of the proposed method *MOOVARM* is higher than the other two methods. We also computed the average values of the classification accuracies of the top 10 rules which were 85.08% (±0.03), 81.96% (±0.02), and 81.65% (±0.02) for the methods, *MOOVARM*, *Apriori* and *Eclat*, respectively (in Figure 4). In addition, the AUC values in terms of sensitivity vs. specificity for classifying the respective disease using the gene sets belonging to the topmost two rules by MOOVARM (from Table 3) from the respective dataset were found as 0.926 and 0.920, respectively (in Figure 5). Moreover, in summary, the average AUC values of the top 10 rules of the same dataset using those methods (MOOVARM, Apriori, and Eclat) were 0.909, 0.861, and 0.859, respectively. Herein, we used an open-source R package “pROC” (Robin et al., 2011) to illustrate the ROC and AUC curves as depicted in Figure 5.

**TABLE 3:** Top 10 rules of MOOVARM with their classification accuracy, specificity, sensitivity, and AUC values.

Rule ID	Rule	Avg. classification accuracy (sd)	Avg. specificity (sd)	Avg. sensitivity (sd)	AUC	Std. overall err. rate
1	{STAT3+, TP53- → MAPK3+}	85.38% (±0.0405)	85.38% (±0.0653)	83.84% (±0.0405)	0.926	0.04054202
2	{STAT3+ → MAPK3+}	84.61% (±0.0181)	76.92% (±0.0243)	92.30% (±0.0243)	0.918	0.01813094
3	{JUN+, STAT3+, TP53- → MAPK3+}	89.23% (±0.0324)	89.23% (±0.0324)	89.23% (±0.0397)	0.967	0.03243362
4	{ESR1+ → MAPK3+}	80.76% (±0.0243)	76.92% (±0.0371)	76.15% (±0.0243)	0.83	0.02432521
5	{JUN+, STAT3+ → MAPK3+}	92.3% (±0.0162)	90% (±0.0228)	87.69% (±0.0324)	0.956	0.02432521
6	{TP53- → MAPK3+}	73.84% (±0.0397)	85.38% (±0.0606)	83.84% (±0.0648)	0.771	0.03972291
7	{JUN+, TP53- → MAPK3+}	83.46% (±0.0506)	95.38% (±0.0519)	71.53% (±0.0653)	0.877	0.05063697
8	{ESR1-, MAPK3+ → STAT3+}	84.61% (±0.0268)	83.84% (±0.0371)	85.38% (±0.0371)	0942	0.02689253
9	{MAPK3+, TP53-, AR+ → JUN+}	91.92% (±0.0326)	82.30% (±0.0537)	82.30% (±0.0537)	0.956	0.03268602
10	{STAT3+, MAPK3+ → ESR1-}	84.61% (±0.0268)	83.84% (±0.0371)	85.38% (±0.0371)	0.942	0.02689253
	Average	0.8507	0.8477	0.8538	0.909	0.03085

**sd, standard deviation; “+”denotes upregulated and hypo-methylated genes; “−”represents downregulated and hyper-methylated genes.

**TABLE 4:** Top 10 rules of *Apriori* with their classification accuracy, specificity, sensitivity, and AUC values.

Rule ID	Rule	Avg. classification accuracy (sd)	Avg. specificity (sd)	Avg. sensitivity (sd)	AUC	Std. overall err. rate
1	{STAT3- → GRB2-}	84.23% (±0.0121)	83.84% (±0)	87.69% (±0.0243)	0.878	0.01216261
2	{STAT3-, MAPK3- → GRB2-}	83.46% (±0.0198)	76.92% (±0)	100% (±0.0397)	0.888	0.01986145
3	{MAPK3-, GRB2- → STAT3-}	88.46% (±0.0198)	76.92% (±0)	100% (±0.0397)	0.888	0.01216261
4	{GRB2- → STAT3-}	81.15% (±0.0181)	85.38% (±0.0362)	76.92% (±0)	0.90	0.01813094
5	{ESR1- → MAPK3-}	76.53% (±0.0256)	76.92% (±0.0362)	76.15% (±0.0362)	0.815	0.02564103
6	{STAT3- → MAPK3-}	84.23% (±0.0218)	79.23% (±0.0436)	88.46% (±0)	0.915	0.02183255
7	{STAT3-, GRB2- → MAPK3-}	84.61% (±0.0218)	82.3% (±0.0362)	86.92% (±0.0243)	0.902	0.021832557
8	{GRB2- → MAPK3-}	78.84% (±0.0268)	74.61% (±0.0324)	83.07% (±0.0324)	0.817	0.02689253
9	{ESR1- → GRB2-}	83.07% (±0.0371)	77.69% (±0.0324)	88.46% (±0.0606)	0.826	0.03715738
10	{JUN- → GRB2-}	75% (±0.0373)	65.38% (±0.0243)	87.69% (±0.0648)	0.777	0.0373779
	Average	0.8196	0.7792	0.8754	0.861	0.0233

**sd, standard deviation; “+” denotes upregulated and hypo-methylated genes; “−” represents downregulated and hyper-methylated genes.

**TABLE 5:** Top 10 rules of *Eclat* with their classification accuracy, specificity, sensitivity, and AUC values.

Rule ID	Rule	Avg. classification accuracy (sd)	Avg. specificity (sd)	Avg. sensitivity (sd)	AUC	Std. overall err. rate
1	{STAT3-, MAPK3- → GRB2- }	84.61% (±0.0218)	85.38% (±0.0362)	83.84% (±0.0243)	0.902	0.02183255
2	{STAT3- → GRB2-}	80.76% (±0.0243)	84.61% (±0.0362)	76.92% (±0.0324)	0.897	0.02432521
3	{MAPK3-, GRB2- → STAT3-}	84.61% (±0.0162)	85.38% (±0.0243)	83.84% (±0.0243)	0.903	0.01621681
4	{GRB2- → STAT3-}	81.15% (±0.0181)	85.38% (±0)	76.92% (±0)	0.90	0.01813094
5	{ESR1- → MAPK3-}	76.92% (±0.0256)	79.23% (±0.0362)	77.69% (±0.0362)	0.816	0.02564103
6	{STAT3- → GRB2-}	80.77% (±0.0243)	84.62% (±0.0362)	76.92% (±0.0324)	0.897	0.02432521
7	{STAT3- → MAPK3-}	84.23% (±0.0218)	77.69% (±0.0362)	90.77% (±0)	0.915	0.02183255
8	{GRB2- → MAPK3-}	84.62% (±0.0268)	76.92% (±0.0436)	92.30% (±0.0324)	0.817	0.02689253
9	{ESR1-, GRB2-}	83.46% (±0.0371)	76.15% (±0.0324)	90.77% (±0.0324)	0.826	0.03715738
10	{FYN+ → TP53-}	75.38% (±0.0373)	68.46% (±0.0567)	82.30% (±0.0567)	0.714	0.0373779
	Average	0.8165	0.8038	0.8323	0.859	0.0254

*“+” denotes upregulated and hypo-methylated genes; “−” represents downregulated and hyper-methylated genes.

Furthermore, we performed the KEGG pathway and Gene Ontology (GO) analyses using the participating genes belonging to the top rules generated by MOOVARM, and then we identified the GO-terms with significant *p*-values. Table 6 and Table 7 summarize enrichment results for GO terms: molecular function (GO: MF) and cellular component (GO: CC), respectively, containing the resultant rules of MOOVARM, whereas Table 8 provides the enrichment result for Gene Ontology: biological processing (GO: BP) terms containing the resultant rules of MOOVARM. Table 9 describes the KEGG pathways having the resultant rules of MOOVARM. For example, hsa05161: hepatitis B KEGG pathway (*p*-value = 6.20E-06) contained five genes and eight rules out of the 24 evolved rules obtained from MOOVARM as shown in Table 2. These five genes are *GRB2*, *JUN*, *MAPK3*, *TP53*, and *STAT3*, while these eight rules are {STAT3+ → MAPK3+}, {TP53- → MAPK3+}, {MAPK3+, TP53- → STAT3+}, {STAT3+, TP53- → MAPK3+}, {JUN+, STAT3+ → MAPK3+}, {JUN+, TP53- → MAPK3+}, {JUN+,STAT3+,TP53- → MAPK3+}, and {GRB2- → STAT3-}. Similarly, hsa05200: pathways in cancer (*p*-value = =1.11E-05) consisted of six genes (*AR*, *GRB2*, *JUN*, *MAPK3*, *TP53*, and STAT3) and fifteen evolved rules. In the case of the GO:BP terms, GO:0045893 positive regulation of transcription, DNA-templated (*p*-value = =5.31E-07) was associated with six genes (*AR*, *JUN*, *MAPK3*, *TP53*, *ESR1*, and *STAT3*) and eighteen evolved rules, while for the GO:CC terms, GO:0005654 nucleoplasm (*p*-value = =7.70E-05) was associated with seven genes (*AR*, *GRB2*, *JUN*, *MAPK3*, *TP53*, *ESR1*, and STAT3) and nineteen evolved rules. For GO:MF terms, GO:0008134 transcription factor binding (*p*-value = =2.64E-06) was associated with five genes (*AR*, *JUN*, *TP53*, *ESR1*, and *STAT3*) and two generated rules ({JUN+,AR+ → TP53-} and {TP53-,AR+ → JUN+}).

**TABLE 6:** Gene set enrichment result for Gene Ontology: molecular function (GO: MF) terms containing the resultant rules of MOOVARM.

GO:MF	*p*-value	Gene	Associated rule
GO:0008134 transcription factor binding	2.64E-06	*AR*, *JUN*, *TP53*, *ESR1*, and *STAT3*	{JUN+, AR+ → TP53-} and {TP53-, AR+ → JUN+}
GO:0042802 identical protein binding	3.31E-06	*FYN*, *GRB2*, *JUN*, *TP53*, *ESR1*, and *STAT3*	{GRB2- → STAT3-}, {FYN- → TP53+} and { ESR1-, FYN- → TP53+}
GO:0019899 enzyme binding	4.97E-06	*AR*, *FYN*, *JUN*, *TP53*, and *ESR1*	{JUN+, AR+ → TP53-}, {TP53-, AR+ → JUN+}, {FYN- → TP53+}, and {ESR1-, FYN- → TP53+}
GO:0044212 transcription regulatory region DNA binding	6.68E-05	*AR*, *JUN*, *TP53*, and *STAT3*	{JUN+, AR+ → TP53-} and {TP53-, AR+ → JUN+}
GO:0019903 protein phosphatase binding	2.84E-04	*GRB2*, *TP53*, and *STAT3*	{STAT3+ → MAPK3+}, {TP53- → MAPK3+}, {MAPK3+, TP53- → STAT3+}, ……etc.,*
GO:0003700 transcription factor activity , sequence-specific DNA binding	3.18E-04	*AR*, *JUN*, *TP53*, *ESR1*, and *STAT3*	{JUN+, AR+ → TP53-} and {TP53-, AR+ → JUN+}
GO:0003682 chromatin binding	4.03E-04	*AR*, *JUN*, *TP53*, and *ESR1*	{JUN+, AR+ → TP53-} and {TP53-, AR+ → JUN+}
GO:0043565 sequence-specific DNA binding	9.17E-04	*AR*, *JUN*, *TP53*, and *ESR1*	{JUN+, AR+ → TP53-} and {TP53-, AR+ → JUN+}
GO:0003677 DNA binding	0.002636593	*AR*, *JUN*, *TP53*, *ESR1*, and *STAT3*	{JUN+, AR+ → TP53-} and {TP53-, AR+ → JUN+}
GO:0019901 protein kinase binding	0.00964876	*GRB2*, *TP53*, and *STAT3*	{GRB2- → STAT3-}
GO:0005515 protein binding	0.010325405	*AR*, *FYN*, *GRB2*, *JUN*, *MAPK3*, *TP53*, *ESR1*, and *STAT3*	{STAT3+ → MAPK3+}, {TP53- → MAPK3+}, {MAPK3+, TP53- → STAT3+}, ……etc.,*

**See Supplementary Table S1 for more details.

**TABLE 7:** Gene set enrichment result for Gene Ontology: cellular component (GO: CC) terms containing the resultant rules of MOOVARM.

GO:CC	*p*-value	Gene	Associated rule
GO:0005654 nucleoplasm	7.70E-05	*AR*, *GRB2*, *JUN*, *MAPK3*, *TP53*, *ESR1*, and *STAT3*	{STAT3+ → MAPK3+}, {TP53- → MAPK3+}, {MAPK3+, TP53- → STAT3+} ……etc.,*
GO:0005634 nucleus	2.04E-04	*AR*, *FYN*, *GRB2*, *JUN*, *MAPK3*, *TP53*, *ESR1*, and *STAT3*	{STAT3+ → MAPK3+}, {TP53- → MAPK3+}, {MAPK3+, TP53- → STAT3+}, ……etc.,*
GO:0005829 cytosol	2.13E-04	*AR*, *FYN*, *GRB2*, *JUN*, *MAPK3*, *TP53*, and *STAT3*	{STAT3+ → MAPK3+}, {TP53- → MAPK3+}, {MAPK3+, TP53- → STAT3+}, ……etc.,*

**See Supplementary Table S3 for more details.

**TABLE 8:** Gene set enrichment result for Gene Ontology: biological processing (GO: BP) terms containing the resultant rules of MOOVARM.

GO:BP	*p*-value	Gene	Associated rule
GO:0045893 positive regulation of transcription, DNA-templated	5.31E-07	*AR*, *JUN*, *MAPK3*, *TP53*, *ESR1*, and *STAT3*	{STAT3+ → MAPK3+}, {TP53- → MAPK3+}, {MAPK3+, TP53- → STAT3+}, ……etc.,*
GO:0016032 viral process	3.31E-06	*FYN*, *GRB2*, *TP53*, *MAPK3*, and *STAT3*	{STAT3+ → MAPK3+}, {TP53- → MAPK3+}, {MAPK3+, TP53- → STAT3+}, ……etc.,*
GO:0045944 positive regulation of transcription from RNA polymerase II promoter	1.28E-05	*AR*, *JUN*, *MAPK3*, *TP53*, *ESR1*, and *STAT3*	{STAT3+ → MAPK3+}, {TP53- → MAPK3+}, {MAPK3+, TP53- → STAT3+}, ……etc.,*
GO:0008285 negative regulation of cell proliferation	4.25E-04	*AR*, *JUN*, *TP53*, and *STAT3*	{JUN+, AR+ → TP53-} and {TP53-, AR+ → JUN+}
GO:0007586 aging	0.001951	*GRB2*, *JUN*, and *STAT3*	{GRB2- → STAT3-} and {TP53-, AR+ → JUN+}
GO:0042981 regulation of the apoptotic process	0.003225	*FYN*, *TP53*, and *ESR1*	{FYN- → TP53+} and {ESR1-, FYN- → TP53+}
GO:0006351 transcription, DNA-templated	0.004792	*AR*, *MAPK3*, *TP53*, *ESR1*, and *STAT3*	{STAT3+ → MAPK3+}, {TP53- → MAPK3+}, {MAPK3+, TP53- → STAT3+}, ……etc.,*
GO:0060397 JAK-STAT cascade involved in the growth hormone signaling pathway	0.006237	*MAPK3* and *STAT3*	{STAT3+ → MAPK3+}
GO:0030154 cell differentiation	0.014472	*FYN*, *GRB2*, and *TP53*	{FYN- → TP53+}
GO:0016310 phosphorylation	0.040956	*MAPK3* and *STAT3*	{STAT3+ → MAPK3+}
GO:0006461 protein complex assembly	0.047374	*MAPK3* and *TP53*	{TP53- → MAPK3+}

**See Supplementary Table S4 for more details.

**TABLE 9:** Gene set enrichment result for KEGG pathways containing the resultant rules of MOOVARM.

KEGG pathway	*p*-value	Gene	Associated rule
hsa05161: hepatitis B	6.20E-06	*GRB2*, *JUN*, *MAPK3*, *TP53*, and *STAT3*	{STAT3+ → MAPK3+}, {TP53- → MAPK3+}, {MAPK3+, TP53- → STAT3+}, ……etc.,*
hsa05200: pathways in cancer	1.11E-05	*AR*, *GRB2*, *JUN*, *MAPK3*, *TP53*, and *STAT3*	{STAT3+ → MAPK3+}, {TP53- → MAPK3+}, {MAPK3+, TP53- → STAT3+}, ……etc.,*
hsa05205: proteoglycans in cancer	2.23E-05	*GRB2*, *MAPK3*, *TP53*, *ESR1*, and *STAT3*	{STAT3+ → MAPK3+}, {TP53- → MAPK3+}, {MAPK3+, TP53- → STAT3+}, ……etc.,*
hsa05203: viral carcinogenesis	2.46E-05	*GRB2*, *JUN*, *MAPK3*, *TP53*, and *STAT3*	{STAT3+ → MAPK3+}, {TP53- → MAPK3+}, {MAPK3+, TP53- → STAT3+}, ……etc.,*
hsa04917: prolactin signaling pathway	3.53E-05	*GRB2*, *MAPK3*, *ESR1*, and *STAT3*	{STAT3+ → MAPK3+}, {ESR1+ → MAPK3+}, {GRB2- → STAT3-}, {ESR1-,MAPK3- → STAT3-}, and {STAT3-,MAPK3- → ESR1-}
hsa05215: prostate cancer	6.73E-05	*AR*, *GRB2*, *MAPK3*, and *TP53*	{TP53- → MAPK3+}
hsa04915: estrogen signaling pathway	9.58E-05	*GRB2*, *JUN*, *MAPK3*, and *ESR1*	{ESR1+ → MAPK3+}
hsa04660: T-cell receptor signaling pathway	1.08E-04	*FYN*, *GRB2*, *JUN*, and *MAPK3*	{JUN+, FYN+ → MAPK3+}
hsa04722: neurotrophin signaling pathway	1.70E-04	*GRB2*, *JUN*, *MAPK3*, and *TP53*	{TP53- → MAPK3+} and {JUN+, TP53- → MAPK3+}
hsa04380: osteoclast differentiation	2.20E-04	*FYN*, *GRB2, JUN*, and *MAPK3*	{JUN+, FYN+ → MAPK3+}
hsa05160: hepatitis C	2.31E-04	*GRB2*, *MAPK3*, *TP53*, and *STAT3*	{STAT3+ → MAPK3+}, {TP53- → MAPK3+}, {MAPK3+, TP53- → STAT3+}, {STAT3+, TP53- → MAPK3+}, and {GRB2- → STAT3-}
hsa05213: endometrial cancer	0.00113869	*GRB2*, *MAPK3*, and *TP53*	{TP53- → MAPK3+}
hsa05223: non-small cell lung cancer	0.00131991	*GRB2*, *MAPK3*, and *TP53*	{TP53- → MAPK3+}
hsa05221: acute myeloid leukemia	0.00131991	*GRB2*, *MAPK3*, and *STAT3*	{STAT3+ → MAPK3+} and {GRB2- → STAT3-}
hsa04010: MAPK signaling pathway	0.00155686	*GRB2*, *JUN*, *MAPK3*, and *TP53*	{TP53- → MAPK3+} and {JUN+, TP53- → MAPK3+}
hsa05210: colorectal cancer	0.00161604	*JUN*, *MAPK3*, and *TP53*	{TP53- → MAPK3+} and {JUN+, TP53- → MAPK3+}
hsa05214: glioma	0.00177498	*GRB2*, *MAPK3*, and *TP53*	{TP53- → MAPK3+}
hsa05212: pancreatic cancer	0.00177498	*MAPK3*, *TP53*, and *STAT3*	{STAT3+ → MAPK3+}, {TP53- → MAPK3+}, {MAPK3+, TP53- → STAT3+}, and {STAT3+, TP53- → MAPK3+}
hsa05220: chronic myeloid leukemia	0.0021738	*GRB2*, *MAPK3*, and *TP53*	{TP53- → MAPK3+}
hsa04919: thyroid hormone signaling pathway	0.00536748	*MAPK3*, *TP53*, and *ESR1*	{TP53- → MAPK3+} and {ESR1+ → MAPK3+}
hsa04071: sphingolipid signaling pathway	0.00593268	*FYN*, *MAPK3*, and *TP53*	{TP53- → MAPK3+}
hsa05162: measles	0.00724782	*FYN*, *TP53*, and *STAT3*	{FYN- → TP53+}
hsa04068: FOXO signaling pathway	0.00735406	*GRB2*, *MAPK3*, and *STAT3*	{STAT3+ → MAPK3+}
hsa04550: signaling pathways regulating pluripotency of stem cells	0.0080066	*GRB2*, *MAPK3*, and *STAT3*	{STAT3+ → MAPK3+}
hsa04062: chemokine signaling pathway	0.01384442	*GRB2*, *MAPK3*, and *STAT3*	{STAT3+ → MAPK3+}
hsa05216: thyroid cancer	0.02902286	*MAPK3* and *TP53*	{TP53- → MAPK3+}
hsa05206: microRNAs in cancer	0.03102958	*GRB2*, *TP53*, and *STAT3*	{GRB2- → STAT3-}
hsa05219: bladder cancer	0.04081937	*MAPK3* and *TP53*	{TP53- → MAPK3+}
hsa04151: PI3K-Akt signaling pathway	0.04418067	*GRB2*, *MAPK3*, and *TP53*	{TP53- → MAPK3+}

Association rule mining is related to the directional signature and its effects on disease discovery. The top association rule is STAT3+, TP53- → MAPK3+, where *STAT3* and *TP53* play opposing roles in cellular pathway regulation. According to the literature survey, the activation function of *STAT3* upregulates the survival pathway, whereas p53 activates the apoptotic pathway. *STAT3* contributes to cancer cell proliferation and is associated with tumor malignancy. Similarly, *TP53* is a well-known tumor suppressor gene. *TP53* provides protection against DNA damage by inducing cell cycle arrest, DNA repair, or apoptosis. Mutation of *p53* is often observed in cancer, especially in late events in malignant progression (Pham et al., 2020). The rule says where if antecedent genes (STAT3+, TP53-) are expressed/methylated in a specified manner together, then it is likely that consequent genes (MAPK3+) will also be expressed/methylated in a specified manner together. According to the literature survey, *MARK3* regulates the proliferation and bone metastasis of human breast cancer cells (Du et al., 2020).

To illustrate the efficiency of our top 10 association rules, we conducted literature mining. Our first association rule {STAT3+, TP53- → MAPK3+} says that if antecedent genes (STAT3+, TP53-) are expressed/methylated in a specified manner together, then it is likely that consequent genes (MAPK3+) will also be expressed/methylated in a specified manner together. According to Yang et al. (2021), Yang et al. (2022), and Liu et al. (2022), we obtained these three genes, namely, *STAT3*, *TP53*, and *MAPK3* together as core target genes or highest degree hub genes related to several diseases like gastric cancer and type 2 diabetes mellitus. According to Zu et al. (2021), the three genes of the second association rule, *JUN*, *TP53*, and *MAPK3* together, are related to gastric cancer. According to Santh Rani (2023), the three genes associated with rule 8, *ESR1*, *MAPK3*, and *STAT3* together, are found as the top hub protein target genes in the PPI network analysis. Therefore, we can conclude that the genes associated with our association rules also jointly played several roles in recent literature studies.

However, in the conceptual prospective, the MOOVARM approach modifies the traditional concept of using the static support threshold and a static confidence threshold which were generally applied to maintain these same thresholds across all item sets (i.e., gene sets) in the traditional algorithms like Apriori and Eclat. In MOOVARM, after post-discretization, the association rule mining algorithm utilizes the weighted shortest distance that depended on multiple minimum support thresholds, multiple minimum confidence thresholds, and multiple minimum lift thresholds instead of the static support threshold and the static confidence threshold. Those multiple/dynamic minimum thresholds were estimated by the integration of gene expression, methylation, and protein–protein interaction profiles and a weighted shortest distance-based scheme. The MOOVARM method works on all three different types of profiles: gene expression, methylation, and protein–protein interaction profiles instead of individual datasets like gene expression or DNA methylation or any other data, and produced multi-objective multi-prolific association rules. We also applied a multi-objective optimization technique, TOPSIS, which is named the multi-criteria decision-making technique. It is the procedure to select the best alternative of the set of finite alternatives with respect to multiple criteria. Herein, we ranked the association rules using multiple criteria (such as weighted support, weighted confidence, and weighted lift) and chose the top-ranked association rules through the multi-objective optimization technique. Thus, in a single word, the traditional rule mining algorithms like Apriori and Eclat use static user-defined threshold values and there is no optimized ranking of the estimated rules, while MOOVARM follows dynamic thresholds and generates optimized association rules using multi-objective optimization on various objectives/criteria/rule interestingness values for each individual rule.

Furthermore, to explain this relationship between association rule mining and directional gene signature and its effects on disease discovery, we took the topmost optimized association rule {STAT3+, TP53- → MAPK3+} estimated by MOOVARM (Table 3) for example, which is a directional gene signature. The rule states that if the antecedent genes express and methylate in a specified manner together (i.e., *STAT3* is upregulated and hypo-methylated, and *TP53* is downregulated and hyper-methylated, concurrently), then it is likely that consequent genes will be expressed and methylated in a specified manner together (i.e., MAPK3 will be upregulated and hypo-methylated). The combined effects of the rule create a directional three-gene signature since the total number of the participating genes in the associated rule is three here.

## 6 Conclusion

In this article, we proposed a unique associated rule mining method denoted as *MOOVARM* to find the most acceptable and appropriate rule for multi-omics profiles. To produce the interesting rules for multi-omics profiles, we used and integrated gene expression, methylation, and protein–protein interaction data based on the idea of multi-objective optimization and weighted shortest distance. For this purpose, we identified *PIS* and *NIS* with respect to all gene sets. *PIS* maximized the profit and minimized the loss. Alternatively, *NIS* maximized the loss and minimized the profit. Then, we calculated the distances *d* + and *d* − from *PIS* and *NIS*, respectively, for each gene set. Then, with the help of these two distances, we measured the relative closeness to *PIS* for ranking the gene sets. In this proposed method, we computed relative closeness scores globally instead of individual genes. Finally, MOOVARM generated the final rank of the extracted (multi-objective optimized) rules of correlated genes which may play a significant role in better disease classification performance than the state-of-the-art algorithms in disease discovery as well as therapeutic value. However, the limitation to this work is that MOOVARM works on the multi-omics RNAseq/microarray dataset consisting of DNA methylation, gene expression dataset for the same set of patients/samples, and protein–protein interaction dataset in this framework. MOOVARM cannot work on the single-omics data. Furthermore, our method might not work on single-cell sequencing data without the usage of the matrix imputation prior to pre-filtering steps.

As a future work, we will include more datasets with the advanced added mechanism. In addition, we are interested to further use our proposed model for determining the directional optimized gene signatures in hub gene findings in the multi-molecular regulation study (i.e., the regulation among the long non-coding RNAs, transcription factors, microRNAs, and target genes). Moreover, we also want to use this method in single-cell RNA sequencing and single-cell ATAC sequencing data to detect directional gene signatures for cancer detection.

Furthermore, we have checked some state-of-the-art works of association rule mining based on the fuzzy or rough set theory, but the outcome rules are not good enough, which means that the outcomes are not always beneficial using fuzzy/rough set-based association rule mining (Sharmila and Vijayarani, 2019; Singh and Ganesh Wayal, 2012). Comparatively, as our proposed method MOOVARM used the multi-objective optimization technique, the outcome rules are optimized and efficient enough. Therefore, only the inclusion of the fuzzy/rough set is not always beneficial. Thus, we assume that to improve the performance of the fuzzy/rough theory-based method, the inclusion of multi-objective optimization technique together with fuzzy/rough set-based rule mining will be an efficient step. Therefore, as our future work, we will extend our proposed framework by including both fuzzy/rough theory and multi-objective optimization to produce better and more effective association rule mining from multi-omics data.

## Data Availability

The original contributions presented in the study are publicly available. This data can be found here: https://www.ncbi.nlm.nih.gov/geo/query/acc.cgi?acc=GSE52392
