# Supplementary material for: Optimal ranking and directional signature classification using the integral strategy of multi-objective optimization-based association rule mining of multi-omics data
Source: Front Bioinform. 2023 Jul 27;3:1182176. doi: 10.3389/fbinf.2023.1182176 (PMC10415913; doi:10.3389/fbinf.2023.1182176)
Supplement: Supplementary file 1 [file DataSheet1.PDF]

Table 1. Gene set enrichment result for Gene Ontology: Molecular Function (GO:MF) terms containing the resultant rules of MOOVARM.

GO:MF	P-value	Genes	Associated rules
GO:0008134 transcription factor binding	2.64E-06	AR, JUN, TP53, ESR1, STAT3	{JUN+,AR+→TP53-}, {TP53-,AR+→JUN+}
GO:0042802 identical protein binding	3.31E-06	FYN, GRB2, JUN, TP53, ESR1, STAT3	{GRB2-→STAT3-}, {FYN-→TP53+}, { ESR1-,FYN-→TP53+}
GO:0019899 enzyme binding	4.97E-06	AR, FYN, JUN, TP53, ESR1	{JUN+,AR+→TP53-},{TP53-,AR+→JUN+},{FYN-→TP53+},{ESR1-,FYN-→TP53+}
GO:0044212 transcription regulatory region DNA binding	6.68E-05	AR, JUN, TP53, STAT3	{JUN+,AR+→TP53-}, {TP53-,AR+→JUN+}
GO:0019903 protein phosphatase binding	2.84E-04	GRB2, TP53, STAT3	{STAT3+→MAPK3+}, {TP53-→MAPK3+}, {MAPK3+,TP53-→STAT3+}, {STAT3+,TP53-→MAPK3+}, {ESR1+→MAPK3+}, {JUN+,STAT3+→MAPK3+}, {JUN+,FYN+→MAPK3+}, {STAT3+,FYN+→MAPK3+}, {JUN+,TP53-→MAPK3+}, {JUN+,STAT3+,TP53-→MAPK3+}, {FYN+,TP53-→MAPK3+}, {JUN+,AR+→MAPK3+}, {JUN+,AR+→TP53-}, {TP53-,AR+→JUN+}, {JUN+,AR+→MAPK3+,TP53-}, {MAPK3+,JUN+,AR+ →TP53-},{TP53-,AR+ →MAPK3+,JUN+}, {MAPK3+,TP53-,AR+→JUN+}, {JUN+,TP53-,AR+→MAPK3+}, {GRB2-→STAT3-}, {FYN-→TP53+}, {ESR1-,FYN-→TP53+}, {ESR1-,MAPK3-→STAT3-}, {STAT3-,MAPK3-→ESR1-}
GO:0003700 transcription factor activity, sequence-specific DNA binding	3.18E-04	AR, JUN, TP53, ESR1, STAT3	{JUN+,AR+→TP53-}, {TP53-,AR+→JUN+}
GO:0003682 chromatin binding	4.03E-04	AR, JUN, TP53, ESR1	{JUN+,AR+→TP53-}, {TP53-,AR+→JUN+}
GO:0043565 sequence-specific DNA binding	9.17E-04	AR, JUN, TP53, ESR1	{JUN+,AR+→TP53-}, {TP53-,AR+→JUN+}
GO:0003677 DNA binding	0.002636593	AR, JUN, TP53, ESR1, STAT3	{JUN+,AR+→TP53-}, {TP53-,AR+→JUN+}
GO:0019901 protein kinase binding	0.00964876	GRB2, TP53, STAT3	{GRB2-→STAT3-}
GO:0005515 protein binding	0.010325405	AR, FYN, GRB2, JUN, MAPK3, TP53, ESR1, STAT3	{STAT3+→MAPK3+}, {TP53-→MAPK3+},{MAPK3+,TP53-→STAT3+}, {STAT3+,TP53-→MAPK3+}, {ESR1+→MAPK3+}, {JUN+,STAT3+→MAPK3+}, {JUN+,FYN+→MAPK3+}, {STAT3+,FYN+ →MAPK3+}, {JUN+,TP53-→MAPK3+}, {JUN+,STAT3+,TP53-→MAPK3+}, {FYN+,TP53-→MAPK3+}, {JUN+,AR+→MAPK3+}, {JUN+,AR+→TP53-}, {TP53-,AR+→JUN+}, {JUN+,AR+→MAPK3+,TP53-}, {MAPK3+,JUN+,AR+→TP53-}, {TP53-,AR+→MAPK3+,JUN+}, {MAPK3+,TP53-,AR+→JUN+}, {JUN+,TP53-,AR+→MAPK3+}, {GRB2-→STAT3-}, {FYN-→TP53+}, {ESR1-,FYN-→TP53+}, {ESR1-,MAPK3-→STAT3-}, {STAT3-,MAPK3-→ESR1-}

Table 2. Gene set enrichment result for KEGG pathways containing the resultant rules of MOOVARM.

KEGG pathway	P-value	Genes	Associated rules
hsa05161:Hepatitis B	6.20E-06	GRB2, JUN, MAPK3, TP53, STAT3	{STAT3+→MAPK3+}, {TP53-→MAPK3+}, {MAPK3+,TP53-→STAT3+}, {STAT3+,TP53-→MAPK3+}, {JUN+,STAT3+→MAPK3+}, {JUN+,TP53-→MAPK3+}, {JUN+,STAT3+,TP53-→MAPK3+}, {GRB2-→STAT3-}
hsa05200:Pathways in cancer	1.11E-05	AR, GRB2, JUN, MAPK3, TP53, STAT3	{STAT3+→MAPK3+}, {TP53-→MAPK3+}, {MAPK3+,TP53-→STAT3+}, {STAT3+,TP53-→MAPK3+}, {JUN+,TP53-→MAPK3+}, {JUN+,STAT3+,TP53-→MAPK3+}, {JUN+,AR+→MAPK3+}, {JUN+,AR+→TP53-}, {TP53-,AR+→JUN+}, {JUN+,AR+→MAPK3+,TP53-}, {APK3+,JUN+,AR+→TP53-}, {TP53-,AR+ →MAPK3+,JUN+}, {MAPK3+,TP53-,AR+→JUN+}, {JUN+,TP53-,AR+→MAPK3+}, {GRB2-→STAT3-}
hsa05205: Proteoglycans in cancer	2.23E-05	GRB2, MAPK3, TP53, ESR1, STAT3	{STAT3+→MAPK3+}, {TP53-→MAPK3+}, {MAPK3+,TP53-→STAT3+}, {STAT3+,TP53-→MAPK3+}, {ESR1+→MAPK3+}, {GRB2-→STAT3-}, {ESR1-,MAPK3-→STAT3-}, {STAT3-,MAPK3-→ESR1-}
hsa05203:Viral carcinogenesis	2.46E-05	GRB2, JUN, MAPK3, TP53, STAT3	{STAT3+→MAPK3+}, {TP53-→MAPK3+}, {MAPK3+,TP53-→STAT3+}, {STAT3+,TP53-→MAPK3+}, {JUN+,STAT3+→MAPK3+}, {JUN+,TP53-→MAPK3+}, {JUN+,STAT3+,TP53- →MAPK3+}, {GRB2-→STAT3-}
hsa04917:Prolactin signaling pathway	3.53E-05	GRB2, MAPK3, ESR1, STAT3	{STAT3+→MAPK3+}, {ESR1+→MAPK3+}, {GRB2-→STAT3-}, {ESR1-,MAPK3-→STAT3-}, {STAT3-,MAPK3-→ESR1-}
hsa05215:Prostate cancer	6.73E-05	AR, GRB2, MAPK3, TP53	{TP53-→MAPK3+}
hsa04915:Estrogen signaling pathway	9.58E-05	GRB2, JUN, MAPK3, ESR1	{ESR1+→MAPK3+}
hsa04660:T cell receptor signaling pathway	1.08E-04	FYN, GRB2, JUN, MAPK3	{JUN+,FYN+→MAPK3+}
hsa04722: Neurotrophin signaling pathway	1.70E-04	GRB2, JUN, MAPK3, TP53	{TP53-→MAPK3+}, {JUN+,TP53-→MAPK3+}
hsa04380: Osteoclast differentiation	2.20E-04	FYN, GRB2, JUN, MAPK3	{JUN+,FYN+→MAPK3+}
hsa05160:Hepatitis C	2.31E-04	GRB2, MAPK3, TP53, STAT3	{STAT3+→MAPK3+}, {TP53-→MAPK3+}, {MAPK3+,TP53-→STAT3+}, {STAT3+,TP53-→MAPK3+}, {GRB2-→STAT3-}
hsa05213: Endometrial cancer	0.00113869	GRB2, MAPK3, TP53	{TP53-→MAPK3+}
hsa05223:Non-small cell lung cancer	0.00131991	GRB2, MAPK3, TP53	{TP53-→MAPK3+}
hsa05221:Acute myeloid leukemia	0.00131991	GRB2, MAPK3, STAT3	{STAT3+→MAPK3+}, {GRB2-→STAT3-}
hsa04010:MAPK signaling pathway	0.00155686	GRB2, JUN, MAPK3, TP53	{TP53-→MAPK3+}, {JUN+,TP53-→MAPK3+}
hsa05210: Colorectal cancer	0.00161604	JUN, MAPK3, TP53	{TP53-→MAPK3+}, {JUN+,TP53-→MAPK3+}
hsa05214:Glioma	0.00177498	GRB2, MAPK3, TP53	{TP53-→MAPK3+}
hsa05212:Pancreatic cancer	0.00177498	MAPK3, TP53, STAT3	{STAT3+→MAPK3+}, {TP53-→MAPK3+}, {MAPK3+,TP53-→STAT3+}, {STAT3+,TP53- →MAPK3+}
hsa05220:Chronic myeloid leukemia	0.0021738	GRB2, MAPK3, TP53	{TP53-→MAPK3+}
hsa04919:Thyroid hormone signaling pathway	0.00536748	MAPK3, TP53, ESR1	{TP53-→MAPK3+}, {ESR1+→MAPK3+}
hsa04071:Sphingolipid signaling pathway	0.00593268	FYN, MAPK3, TP53	{TP53-→MAPK3+}
hsa05162:Measles	0.00724782	FYN, TP53, STAT3	{FYN-→TP53+}

KEGG pathway	P-value	Genes	Associated rules
hsa04068:FoxO signaling pathway	0.00735406	GRB2, MAPK3, STAT3	{STAT3+→MAPK3+}
hsa04550:Signaling pathways regulating pluripotency of stem cells	0.0080066	GRB2, MAPK3, STAT3	{STAT3+→MAPK3+}
hsa04062:Chemokine signaling pathway	0.01384442	GRB2, MAPK3, STAT3	{STAT3+→MAPK3+}
hsa05216:Thyroid cancer	0.02902286	MAPK3, TP53	{TP53-→MAPK3+}
hsa05206:MicroRNAs in cancer	0.03102958	GRB2, TP53, STAT3	{GRB2-→STAT3-}
hsa05219:Bladder cancer	0.04081937	MAPK3, TP53	{TP53-→MAPK3+}
hsa04151:PI3K-Akt signaling pathway	0.04418067	GRB2, MAPK3, TP53	{TP53-→MAPK3+}

Table 3. Gene set enrichment result for Gene Ontology: Cellular Component (GO:CC) terms containing the resultant rules of MOOVARM.

GO:CC	P-value	Genes	Associated rules
GO:0005654 nucleoplasm	7.70E-05	AR, GRB2, JUN, MAPK3, TP53, ESR1, STAT3	{STAT3+→MAPK3+}, {TP53-→MAPK3+}, {MAPK3+,TP53-→STAT3+},{JUN+,TP53-→MAPK3+}, {STAT3+,TP53-→MAPK3+}, {JUN+,AR+→TP53-}, {ESR1+→MAPK3+}, {JUN+,STAT3+→MAPK3+}, {JUN+,STAT3+,TP53-→MAPK3+}, {JUN+,AR+→MAPK3+}, {TP53-,AR+→JUN+}, {JUN+,AR+→MAPK3+,TP53-}, {MAPK3+,JUN+,AR+→TP53-}, {TP53-,AR+→ MAPK3+,JUN+}, {MAPK3+,TP53-,AR+→JUN+}, {JUN+,TP53-,AR+→ MAPK3+}, {GRB2-→STAT3-}, {ESR1-,MAPK3-→STAT3-}, {STAT3-,MAPK3-→ESR1-}
GO:0005634 nucleus	2.04E-04	AR, FYN, GRB2, JUN, MAPK3, TP53, ESR1, STAT3	{STAT3+→MAPK3+}, {TP53-→MAPK3+}, {MAPK3+,TP53-→STAT3+}, {STAT3+,TP53-→MAPK3+}, {ESR1+→MAPK3+}, {JUN+,STAT3+→MAPK3+}, {JUN+,FYN+→MAPK3+}, {STAT3+,FYN+→MAPK3+}, {JUN+,TP53-→MAPK3+}, {JUN+,STAT3+,TP53-→MAPK3+}, {FYN+,TP53-→MAPK3+}, {JUN+,AR+→MAPK3+}, {JUN+,AR+→TP53-}, {TP53-,AR+→JUN+}, {JUN+,AR+→MAPK3+,TP53-}, {MAPK3+,JUN+,AR+→TP53-}, {TP53-,AR+→MAPK3+,JUN+}, { MAPK3+,TP53-,AR+→JUN+}, {JUN+,TP53-,AR+→MAPK3+}, {GRB2-→STAT3-}, {FYN-→ TP53+}, { ESR1-,FYN-→TP53+}, {ESR1-,MAPK3-→STAT3-}, {STAT3-,MAPK3-→ESR1-}
GO:0005829 cytosol	2.13E-04	AR, FYN, GRB2, JUN, MAPK3, TP53, STAT3	{STAT3+→MAPK3+}, {TP53-→MAPK3+}, {MAPK3+,TP53-→STAT3+}, {STAT3+,TP53-→MAPK3+}, {JUN+,STAT3+→MAPK3+}, {JUN+,FYN+→MAPK3+}, {STAT3+,FYN+→MAPK3+}, {JUN+,TP53-→MAPK3+}, {JUN+,STAT3+,TP53-→ MAPK3+}, {FYN+,TP53-→MAPK3+}, {JUN+,AR+→MAPK3+}, {JUN+,AR+→TP53-}, {TP53-,AR+→JUN+}, {JUN+,AR+→MAPK3+,TP53-}, {MAPK3+,JUN+,AR+→TP53-}, {TP53-,AR+→MAPK3+,JUN+}, {MAPK3+,TP53-,AR+→JUN+}, {JUN+,TP53-,AR+→MAPK3+}, {GRB2-→STAT3-}, {FYN-→TP53+}

Table 4. Gene set enrichment result for Gene Ontology: Biological Processing (GO:BP) terms containing the resultant rules of MOOVARM.

GO:BP	P-value	Gene	Associated rules
GO:0045893 positive regulation of transcription, DNA-templated	5.31E-07	AR, JUN, MAPK3, TP53, ESR1, STAT3	{STAT3+→MAPK3+}, {TP53-→MAPK3+}, {MAPK3+,TP53-→STAT3+}, {JUN+,AR+→TP53-}, {JUN+,STAT3+→MAPK3+}, {STAT3+,TP53-→MAPK3+}, {ESR1+→MAPK3+}, {JUN+,TP53-→MAPK3+}, {JUN+,STAT3+,TP53-→MAPK3+}, {JUN+,AR+→MAPK3+}, {TP53-,AR+→JUN+}, {JUN+,AR+→MAPK3+,TP53-}, {MAPK3+,JUN+,AR+→TP53-}, {TP53-,AR+ →MAPK3+,JUN+}, {MAPK3+,TP53-,AR+→JUN+}, {JUN+,TP53-,AR+→MAPK3+}, {ESR1-,MAPK3-→STAT3-}, {STAT3-,MAPK3-→ESR1-}
GO:0016032 viral process	3.31E-06	FYN, GRB2, TP53, MAPK3, STAT3	{STAT3+→MAPK3+}, {TP53-→MAPK3+}, {MAPK3+,TP53-→STAT3+}, {STAT3+,TP53-→MAPK3+}, {STAT3+,FYN+→MAPK3+}, {FYN+,TP53-→MAPK3+}, {GRB2-→STAT3-}, {FYN-→TP53+}
GO:0045944 positive regulation of transcription from RNA polymerase II promoter	1.28E-05	AR, JUN, MAPK3, TP53, ESR1, STAT3	{STAT3+→MAPK3+}, {TP53-→MAPK3+}, {MAPK3+,TP53-→STAT3+}, {STAT3+,TP53-→MAPK3+}, {ESR1+→MAPK3+}, {JUN+,STAT3+→MAPK3+}, {JUN+,TP53-→MAPK3+}, {JUN+,STAT3+,TP53-→MAPK3+}, {JUN+,AR+→MAPK3+}, {JUN+,AR+→TP53-}, {TP53-,AR+→JUN+}, {JUN+,AR+→MAPK3+,TP53-}, {MAPK3+,JUN+,AR+→TP53-}, {TP53-,AR+→MAPK3+,JUN+}, {MAPK3+,TP53-,AR+→JUN+}, {JUN+,TP53-,AR+→MAPK3+}, {ESR1-,MAPK3-→STAT3-}, {STAT3-,MAPK3-→ESR1-}
GO:0008285 negative regulation of cell proliferation	4.25E-04	AR, JUN, TP53, STAT3	{JUN+,AR+→TP53-}, {TP53-,AR+→JUN+}
GO:0007586 aging	0.001951	GRB2, JUN, STAT3	{GRB2-→STAT3-}, {TP53-,AR+→JUN+}
GO:0042981 regulation of apoptotic process	0.003225	FYN, TP53, ESR1	{FYN-→TP53+}, {ESR1-,FYN-→TP53+}
GO:0006351 transcription, DNA-templated	0.004792	AR, MAPK3, TP53, ESR1, STAT3	{STAT3+→MAPK3+}, {TP53-→MAPK3+}, {MAPK3+,TP53-→STAT3+}, {STAT3+, TP53-→MAPK3+}, {ESR1+→MAPK3+}, {ESR1-,MAPK3-→STAT3-}, {STAT3-,MAPK3-→ESR1-}
GO:0060397 JAK-STAT cascade involved in growth hormone signaling pathway	0.006237	MAPK3, STAT3	{STAT3+→MAPK3+}
GO:0030154 cell differentiation	0.014472	FYN, GRB2, TP53	{FYN-→TP53+}
GO:0016310 phosphorylation	0.040956	MAPK3, STAT3	{STAT3+→MAPK3+}
GO:0006461 protein complex assembly	0.047374	MAPK3, TP53	{TP53-→MAPK3+}
